# Supplementary material for: Molecular Characterization, Expression, and Regulatory Signal Pathway Analysis of Inflammasome Component Apoptosis-Associated Speck-like Protein Containing a CARD Domain (ASC) in Large Yellow Croaker (Larimichthys crocea)
Source: Int J Mol Sci. 2023 Jan 22;24(3):2175. doi: 10.3390/ijms24032175 (PMC9917028; doi:10.3390/ijms24032175)
Supplement: Supplementary file 1 [file ijms-24-02175-s001.zip › ijms-2111304-supplementary.pdf]

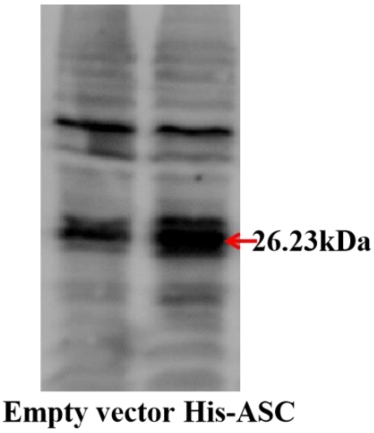

Figure S1. The exogenous expression of LcASC protein in large yellow croaker kidney cells confirmed by Western blot using anti-His antibody.

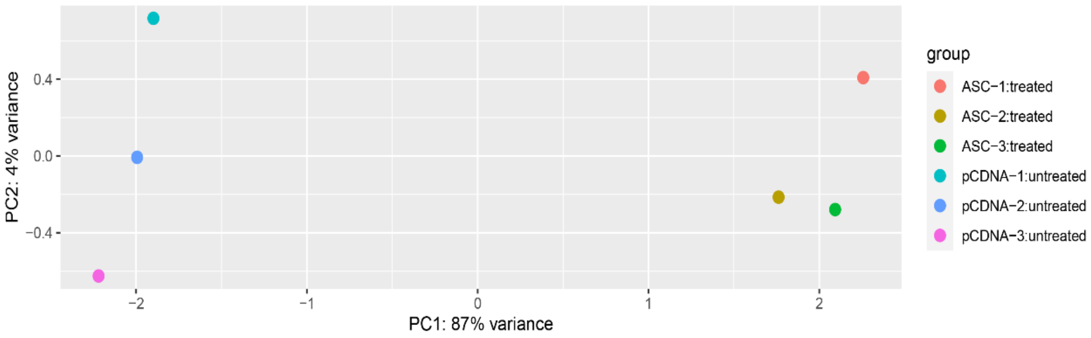

Figure S2. The PCA analysis of all surveyed samples.

Table S1. Primers used for ASC cDNA identification and gene expression.

Primer	Sequence (5'-3')	Purpose
cASC-F	ATGGCCCCCAAAACAAAAAG	Gene identification
cASC-R	TTTTTTATTCTTGATGTCGTCAATG	
qASC-F	CCCCCAAAACAAAAAGAAGGTG	qPCR
qASC-R	CTTTTATCCTTCACCATGGCG	
βactin-F	TTATGAAGGCTATGCCCTGCC	
βactin-R	TGAAGGAGTAGCCACGCTCTGT	
sASC-F	CtaccggactcagatctcgagATGGCCCCCAA AACAAAAAG	Subcellular localization
sASC-R	GtaccgtcgactgcagaattcTTTTTTATTCTTG ATGTCGTCAATG	
oASC-F	CctctagactcgagcggccgcATGGCCCCCAAA ACAAAAAG	Over expression
oASC-R	TagtccagtgtggtggaattcTTTTTTATTCTTG ATGTCGTCAATG	

Table S2. Differently expressed genes between ASC overexpression and control cells.

GeneID	baseMean	log_2FoldChange	lfcSE	stat	pvalue	padj
KIAA0754	66.40229	2.575797	0.301724	8.536926	1.38E-17	4.20E-15
POMK	93.01342	1.761321	0.241924	7.280481	3.33E-13	4.82E-11
NCR3LG1	394.1375	1.522483	0.114191	13.33278	1.49E-40	8.65E-37
FOS	110.2471	1.21052	0.210085	5.762042	8.31E-09	4.91E-07
TSIX	139.6885	1.1931	0.175151	6.81183	9.64E-12	9.97E-10
SLC2A3	546.8132	1.153975	0.087924	13.12471	2.38E-39	9.18E-36
LBH	142.5221	1.077569	0.191819	5.617622	1.94E-08	1.02E-06
XIST	14754.1	0.988873	0.067755	14.59479	3.03E-48	3.51E-44
MAFB	95.00339	0.936685	0.217955	4.2976	1.73E-05	0.00037
GEM	119.0044	0.911408	0.191778	4.752409	2.01E-06	6.00E-05
SYT2	89.84213	0.893659	0.217715	4.104723	4.05E-05	0.000734
SEMA6B	129.4587	0.843701	0.177921	4.742006	2.12E-06	6.27E-05
BHLHE40	980.463	0.841318	0.075729	11.10955	1.13E-28	1.19E-25
ARRDC4	396.5321	0.792961	0.109546	7.238585	4.53E-13	6.40E-11
NDRG1	1120.804	0.770508	0.075006	10.27266	9.36E-25	6.38E-22
MFSD4B	205.2744	0.760713	0.166033	4.581688	4.61E-06	0.000119
SAT1	232.3796	0.756776	0.135847	5.570786	2.54E-08	1.32E-06
RNF169	773.6177	0.752826	0.074929	10.0472	9.45E-24	5.48E-21
EGR1	136.1017	0.731437	0.176253	4.149935	3.33E-05	0.000624
PLEKHM1P1	162.1855	0.727663	0.161959	4.492881	7.03E-06	0.000169
SULF1	624.6119	0.70592	0.097541	7.237182	4.58E-13	6.40E-11
USP37	498.4564	0.701755	0.095065	7.381857	1.56E-13	2.32E-11
AHNAK	1264.461	0.678233	0.058185	11.65657	2.12E-31	2.74E-28
MED12L	198.5478	0.654666	0.148421	4.410874	1.03E-05	0.000231
ZC3H6	220.952	0.651329	0.144934	4.493976	6.99E-06	0.000168
DNHD1	731.569	0.649253	0.076652	8.470111	2.45E-17	7.10E-15
AHNAK2	244.2486	0.613215	0.133164	4.604971	4.13E-06	0.000109
EOLA1	564.2075	0.605084	0.094305	6.416279	1.40E-10	1.16E-08
HEG1	455.3894	0.595556	0.09555	6.23295	4.58E-10	3.47E-08
OSTN	731.8564	0.592394	0.077159	7.677616	1.62E-14	2.80E-12
CPEB2	267.4966	0.587332	0.125794	4.669005	3.03E-06	8.34E-05
EGFR	351.5324	0.577759	0.117019	4.937311	7.92E-07	2.68E-05
ADAMTS1	2776.272	0.577173	0.045331	12.73229	3.91E-37	9.07E-34
GPR50	363.4001	0.563776	0.122966	4.584824	4.54E-06	0.000118
TENM1	2022.592	0.56237	0.062328	9.022723	1.83E-19	6.64E-17
HERC2	545.9844	0.555566	0.093224	5.959487	2.53E-09	1.67E-07
KIF5C	290.1017	0.548832	0.119145	4.606402	4.10E-06	0.000108
HMOX1	788.7589	0.540671	0.093803	5.763908	8.22E-09	4.88E-07
INSIG1	2815.258	0.534416	0.048154	11.098	1.28E-28	1.24E-25
KLHL24	345.3	0.529627	0.112222	4.719454	2.36E-06	6.77E-05
PLEKHH2	286.2567	0.526425	0.123927	4.247876	2.16E-05	0.000443
TSC22D3	4102.144	0.523403	0.043183	12.12046	8.23E-34	1.19E-30
DDR2	2244.403	0.520231	0.044789	11.61511	3.45E-31	4.00E-28
NEAT1	2495.875	0.514695	0.054571	9.431748	4.03E-21	1.95E-18
DOK3	329.3064	-0.58951	0.11167	-5.27905	1.30E-07	5.59E-06
RNA28SN5	247.6619	-0.72932	0.15172	-4.807	1.53E-06	4.75E-05

TableS3. Enriched genes in each pathway.

ID	Description	GeneRa-tio	p.adjust	qvalue	geneID
hsa04928	Parathyroid hormone synthesis, secretion and action	4/22	0.0186	0.0163	EGFR/EGR1/MAFB/FOS
hsa01522	Endocrine resistance	3/22	0.1236	0.1078	EGFR/NOTCH2/FOS
hsa05132	Salmonella infection	4/22	0.1498	0.1307	AHNAK/KIF5C/AHNAK2/FOS
hsa04216	Ferroptosis	2/22	0.1498	0.1307	HMOX1/SAT1
hsa05224	Breast cancer	3/22	0.1547	0.1350	EGFR/NOTCH2/FOS
hsa05223	Non-small cell lung cancer	2/22	0.2346	0.2047	EGFR/KIF5C
hsa05207	Chemical carcinogenesis - receptor activation	3/22	0.2346	0.2047	EGFR/NOTCH2/FOS
hsa05208	Chemical carcinogenesis - reactive oxygen species	3/22	0.2346	0.2047	EGFR/HMOX1/FOS
hsa05210	Colorectal cancer	2/22	0.2346	0.2047	EGFR/FOS
hsa05235	PD-L1 expression and PD-1 checkpoint pathway in cancer	2/22	0.2346	0.2047	EGFR/FOS
hsa04658	Th1 and Th2 cell differentiation	2/22	0.2346	0.2047	NOTCH2/FOS
hsa04912	GnRH signaling pathway	2/22	0.2346	0.2047	EGFR/EGR1
hsa05231	Choline metabolism in cancer	2/22	0.2346	0.2047	EGFR/FOS
hsa04933	AGE-RAGE signaling pathway in diabetic complications	2/22	0.2346	0.2047	FN1/EGR1
hsa04066	HIF-1 signaling pathway	2/22	0.2566	0.2239	EGFR/HMOX1
hsa04919	Thyroid hormone signaling pathway	2/22	0.2715	0.2369	NOTCH2/MED12L
hsa04926	Relaxin signaling pathway	2/22	0.2715	0.2369	EGFR/FOS
hsa04728	Dopaminergic synapse	2/22	0.2715	0.2369	KIF5C/FOS
hsa05206	MicroRNAs in cancer	3/22	0.2715	0.2369	EGFR/HMOX1/NOTCH2
